# Digital anatomical measurements of safe screw placement at superior border of the arcuate line for acetabular fractures

**DOI:** 10.1186/s12891-015-0518-1

**Published:** 2015-03-15

**Authors:** Xiaoxi Ji, Chun Bi, Fang Wang, Yuchen Jiang, Dongmei Wang, Qiugen Wang

**Affiliations:** Trauma Center, Shanghai First People’s Hospital, Shanghai Jiao Tong University, 650 xinsongjiang Rd, Songjiang District, Shanghai 201620 China; School of Mechanical Engineering, Shanghai Jiao Tong University, Shanghai, China

**Keywords:** Acetabular fracture, Screw placement, Arcuate line, Complication, Digital measurement

## Abstract

**Background:**

Screw penetration into hip joint is a severe complication during acetabular fracture surgery, which might result in osteoarthritis and chondrolysis. The purpose of this study was to obtain the safe and effective screw angles and lengths at acetabular area of the fixation route along the superior border of the arcuate line.

**Methods:**

A total of 98 uninjured pelvises of Chinese adults were examined. Each person’s computed tomography (CT) scans were reconstructed to create a three-dimensional pelvic model. A curve of the fixation route was delineated and five cross-sections from the pubic tubercle to the sacroiliac joint direction were constructed perpendicularly to the curve. The minimum safe direction, which was tangent to the acetabulum, was measured in the middle three sections and then recorded as the angle α. The maximum effective direction, which was determined by a 14 mm arc and the quadrilateral surface, was also measured in the above sections and then recorded as the angle β. The maximum screw lengths for the five sections were measured.

**Results:**

The ranges of safe and effective screw insertion angles for the 2nd, 3rd, 4th cross-sections were 21.09±13.57°~40.45±13.60°, 30.43±14.05°~47.54±12.67°, 23.84±11.60°~37.13±8.45°, respectively. The maximum screw lengths for the five sections were 15.89±3.80 mm, 58.83±27.66 mm, 42.94±22.41 mm, 72.43±6.73 mm, 40.99±6.33 mm. The male group showed significantly greater minimum safe angle compared to the female group in the 2nd, 3rd, and 4th sections (p<0.05).

**Conclusions:**

The screw insertion at the acetabular area for the female requires greater minimum safe angle towards the quadrilateral surface than the male.

## Background

The diagnosis, classification, reduction and fixation of acetabular fracture have been regarded difficult for its deep anatomical location and complicated surroundings. The injuries of articular surface increase the incidence of complications, such as malunion and traumatic osteoarthritis, to 50% - 60%, which greatly affect the patients’ life quality [1-3]. As anatomical reduction plays an important role in the clinical outcome and the incidence of complications, the internal fixation with screw-plate system or screws only is getting more and more accepted by orthopedic surgeons [4]. During the procedure of acetabular fixation via ilioinguinal or Stoppa approach, screws might be placed at a wrong direction and penetrate the hip joint since the articular surfaces cannot be observed during the procedure. This can result in severe complications of aggravated osteoarthritis and chondrolysis in the long run [5]. Several methods, including intraoperative radiographs or fluoroscopy, auscultation of the hip with movement, and direct observation of the hip joint, have been reported to prevent this complication. These techniques however increase operation time and iatrogenic trauma [4,6,7]. Only with deep understanding of the unique three dimensional structure of acetabular area, can orthopedic surgeons place screws safely and accurately to avoid the screw penetration of the hip joint [8,9]. It is worth noting that the previous researches obtained the safe paths and safe angles by measurements on cadaveric bone [8,10], which contains a certain level of artificial error during the projected area of acetabulum, the determination of the cross-section, the construction of the supplementary lines, etc. Besides, the sample sizes of these researches are relatively low, ranging from 30 to 46, which are relatively small to evaluate the general situation of population. Digital three-dimensional (3D) measurements, based on CT reconstruction, have the same accuracy and reliability as traditional measurements, and this 3D technology has been considered a more efficient method for orthopaedic anatomic studies, design, and optimization of implants [11-13].

A retrospective observational study by digital reconstruction and 3D measurements of large samples of normal adult pelvic CT scans was conducted by the authors. The aim of this study was to obtain the safe and effective screw angles and lengths at acetabular area of the fixation route along the superior border of the arcuate line. We hypothesized that screw placement at acetabular area was applicable within appropriate direction.

## Methods

### Samples and equipment

We collected the computed tomography (CT) scans from out-patients with varicose vein of lower limb, who needed enhanced CT scans from pelvis to feet for further evaluation and treatments. Each patient was eliminated pelvic deformity, trauma, tumor and other diseases by CT results and inquesting history. From December 2009 to November 2010, 98 complete pelvic CT scans of unrelated ethnic Han Chinese adults (mean age 60.1 (22–91) years, 60 men, 38 women) were collected from the medical image database of the department of radiology of the authors’ institution. During CT scanning, patients kept the standard anatomical horizontal position with lower limbs unbent. All the CT scans were performed at 120 kV and 300 mA with a slice thickness of 0.75 mm by a 64-channel CT, Light Speed VCT XTe (GE Healthcare; Milwaukee, WI, USA), and the scanning time of each slice was 200 ms. There were approximately 380 DICOM format CT images for the pelvic part of each patient. Because the data was collected from out-patients retrospectively, the height and weight of the patient were not recorded in this study. The study was performed with the help of the following software: the interactive medical image control system MIMICS 13.0, the reverse engineering software Geomagics 10, the engineering design software Imageware 23.0, and the mechanical design software Unigraphics NX 7.0. The study was approved by the Institutional Review Board of Shanghai First People’s Hospital.

### Measurement of parameters

DICOM formatted CT scan images of each patient were imported to Mimics 13.0. After removing the soft tissue by thresholding segmentation, region growing, and smoothing process, an entire 3D digital pelvic model was established and saved in Stereo Lithography (STL) format.

The pelvic model was imported to the Geomagics software as a mesh model in the STL format. The horizontal, coronal and sagittal planes were determined in the first place. According to the definition of the anatomical position, the pubic tubercle and anterior superior iliac spine were in the same coronal plane. The plane through the midpoint of the pubic symphysis, the midpoint of the anterior border of the sacral promontory and the coccyx tip was identified the sagittal plane. And the plane constructed perpendicularly to the sagittal and the coronal plane was the horizontal plane. Along with the route from the pubic tubercle, pubis pecten, iliopubic eminence, arcuate line, and sacroiliac joint, more than 15 points at the cortical surface were picked 5.0 mm lateral and superior to the pelvic brim to draw the space curve of the bone surface. All the objects were saved in STL format and imported to the Imageware software, by which an optimal ball was constructed to fit the acetabular fossa. And all the subjects were saved in IMW format and imported to the Unigraphic NX.

**Figure 1:**
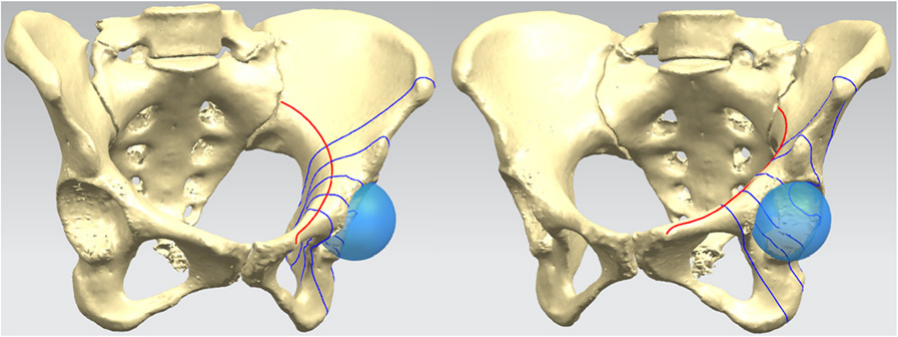
**Five acetabular area sections were obtained by intersecting five normal planes perpendicular to the curve and the pelvis.**

**Figure 2:**
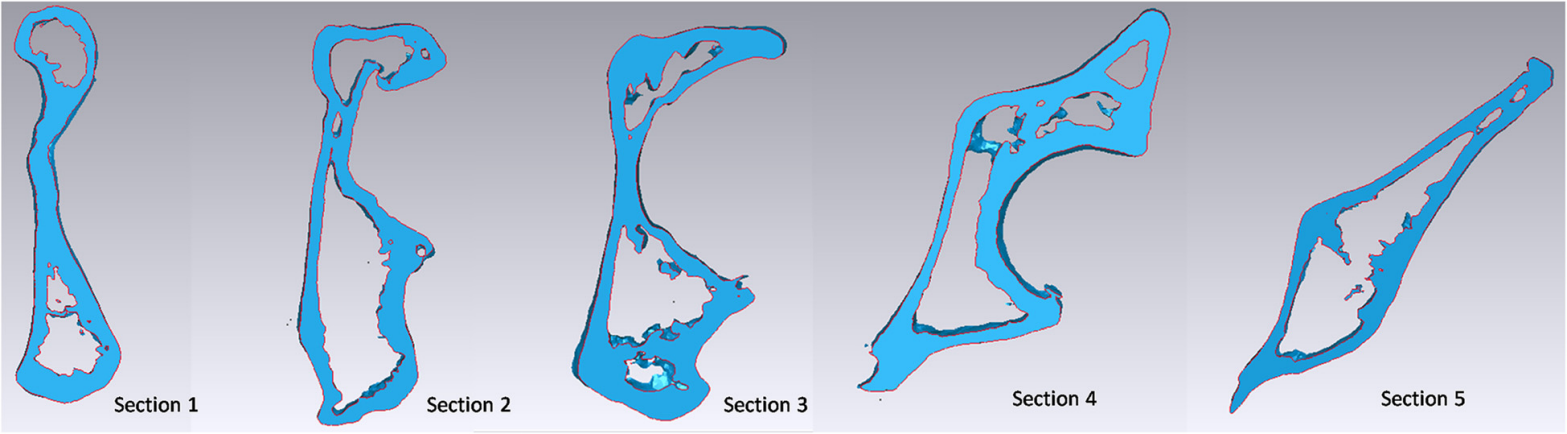
**The five sections were recorded as section 1, 2, 3, 4, and 5 from the pubic tubercle to the sacroiliac joint direction.**

A cross-section through the ball center was made perpendicularly to the aforesaid curve. Two planes parallel to the cross-section forwardly and backwardly, respectively, along the curve by half of radius of ball intersected the space curve at four points. These four points, along with the ball center point, divided the space curve into four equal parts. Five acetabular area sections were obtained by intersecting the previous five parallel planes and the pelvis, respectively (Figure 1). The five sections were recorded as section 1, 2, 3, 4, and 5 from the pubic tubercle to the sacroiliac joint (Figure 2). The intersection point between each cross-section and the space curve was the screw entrance point at this section.

**Figure 3:**
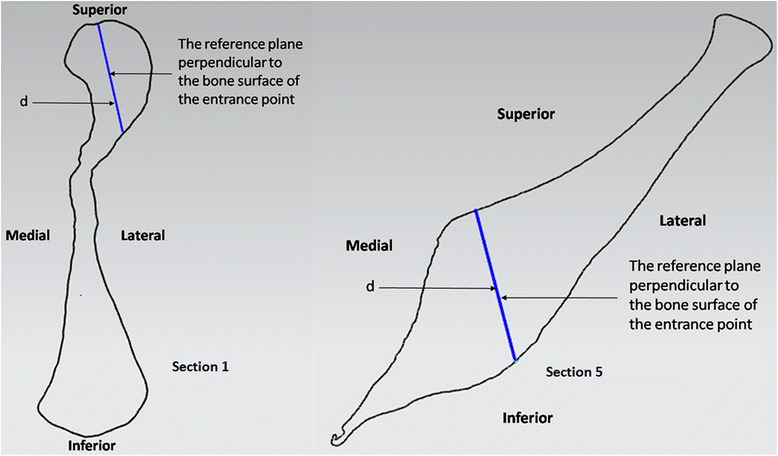
**To section 1 and 5, the distance between the screw entrance point and the intersection point was measured and recorded as the length of the screw, d.**

**Figure 4:**
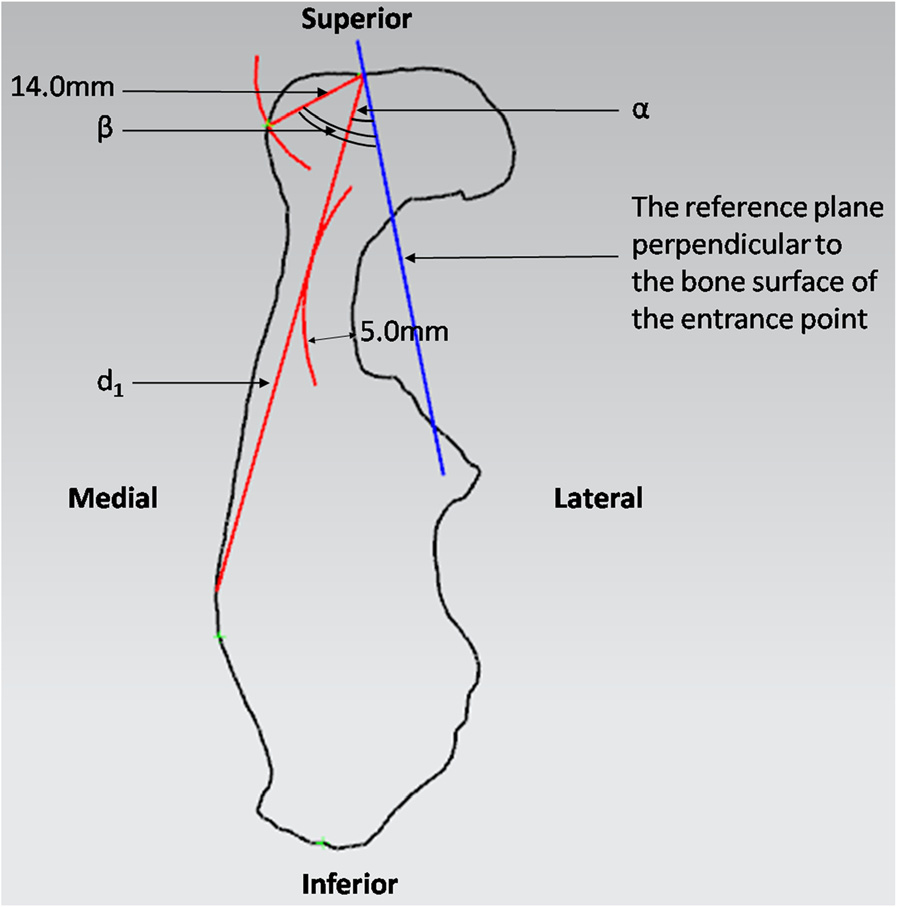
**To section 2 and 3, the line tangent to the expanded arc through the screw entrance point was recorded as the length of the screw, d**_**1**_ **.** The angle between the tangent line and the normal plane was recorded as α. The angle between the line connecting the screw entrance point and the intersection point and the normal plane was recorded as β.

**Figure 5:**
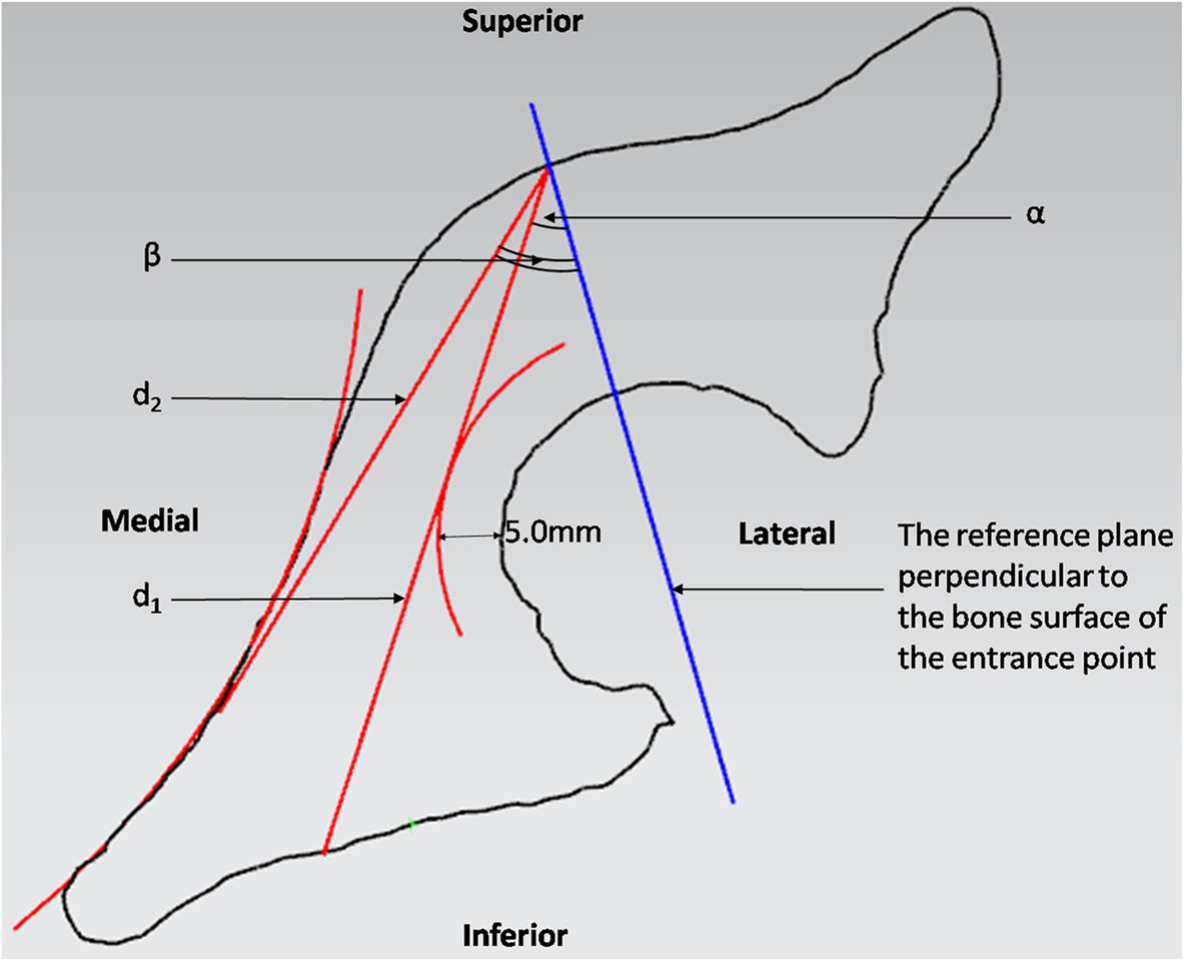
**To section 4, the length of screw d**_**1**_ **and the angleαwere measured with the method same as section 2 and 3.** The line tangent to the arc of quadrilateral surface through the screw entrance was recorded as the length of screw, d_2_. The angle between the tangent line and the normal plane was recorded as β.

At each cross-section, a normal plane was constructed perpendicularly to the bone surface through the screw entrance point and intersected the section at an intersection point. To section 1 and 5, the distance between the screw entrance point and the intersection point was measured and recorded as the length of the screw, d (Figure 3). Sections 2 and 3 were measured with the same method because of similar shapes. An arc was fitted by picking several points along the edge of acetabulum. The radius of the arc was expanded by 5.0 mm to represent the thickness of subchondral bone. The line tangent to the expanded arc through the screw entrance point was recorded as the length of the screw, d_1_. The angle between the tangent line and the normal plane was recorded as α. Another arc with the radius of 14.0 mm was constructed and then intersected with the quadrilateral surface at a point. And the angle between the line connecting the screw entrance point and the intersection point and the normal plane was recorded as β (Figure 4). To section 4, the length of screw d_1_ and the angle α were measured with the method same as section 2 and 3. An arc was fitted by picking several points at the concave of the quadrilateral surface. The line tangent to the arc through the screw entrance was recorded as the length of screw, d_2_. The angle between the tangent line and the normal plane was recorded as β (Figure 5).

### Statistical analysis

The data were expressed as mean±standard deviation and then analyzed by using the descriptive methods with SPSS 19.0. The data distributions were analyzed with normality tests. A *P* value less than 0.05 was considered to be statistically significant.

All 98 pelvises were categorized by gender. We compared the mean values of the relative parameters between different groups with *t*-test.

## Results

The safe screw lengths of all the five cross-sections were 15.89±3.80 mm, 58.83±27.66 mm, 42.94±22.41 mm, 72.43±6.73 mm and 40.99±6.33 mm (Table 1). The ranges of safe and effective screw insertion angles for section 2, 3, 4 were 21.09±13.57°~40.45±13.60°, 30.43±14.05°~47.54±12.67°, 23.84±11.60°~37.13±8.45°.

**Table 1:** **Comparison between male and female**

	**Section 1**	**Section 2**			**Section 3**			**Section 4**				**Section 5**
	**d (mm)**	**d**_**1**_ **(mm)**	**α (°)**	**β (°)**	**d**_**1**_ **(mm)**	**α (°)**	**β (°)**	**d**_**1**_ **(mm)**	**α (°)**	**d**_**2**_ **(mm)**	**β (°)**	**d (mm)**
Total	15.89±3.80	58.83±27.66	21.09±13.57	40.45±13.60	42.94±22.41	30.43±14.05	47.54±12.67	64.30±13.19	23.84±11.60	72.43±6.73	37.13±8.45	40.99±6.33
Male	15.60±4.15	66.38±27.91	18.13±10.41	39.22±12.13	48.27±23.30	26.67±11.60	46.31±12.05	66.39±12.02	21.60±11.02	73.98±6.71	37.94±7.93	41.87±6.41
Female	16.34±3.18	47.27±23.24	25.61±16.48	42.33±15.59	34.64±18.36	36.31±15.63	49.50±13.55	61.10±14.43	27.50±11.75	69.65±5.92	35.68±9.29	39.59±6.03
P value	0.335	0.002	0.022	0.301	0.002	0.001	0.232	0.088	0.017	0.011	0.244	0.081

The comparisons between male and female showed significant differences for d_1_ of section 2, 3, α of section 2, 3, 4, d_2_ of section 4 (*P*<0.05).

## Discussion

The results of this anatomical measurement provided the references for screw placement of acetabular fractures, including the minimum effective angle, maximum safe angle and screw length. In addition, the safe and effective angles were presented with respect to the bone surface, which would facilitate screw placement for clinical applications. Some related studies focusing on screw placement of anterior and posterior risky region of acetabulum were reported in previous literature. Benedetti sectioned cadaveric specimens at 1.0-cm intervals, beginning at the level of the inferior border of the acetabulum (the junction between the anteroinferior edge of the acetabulum and the most anterolateral edge of the superior ramus of the pubic bone). The plane of the cross-section was perpendicular to the anterior column. For the entrance points which were 0.5 cm lateral to the pelvic brim, and 1.0 cm, 2.0 cm, 3.0 cm superior to the starting level respectively, the safe medial angulations were 24.9 ± 4.4°, 29.2 ± 5.5°, 20.7 ± 4.3°, with respect to the perpendicular of the longitudinal axis of the anterior column without penetration of the hip joint [8]. However, the relative position between the longitudinal axis and the screw entrance point at fracture fragments might be changed when suffering from acetabular fractures. Consequently, the angulations this study provided might not be reliable for screw placement during clinical surgeries. The results of our study were correspondent with Benedetti’s study, except that a different reference plane was used. The angulations of our study were related to the bone surface other than the longitudinal axis, which could guarantee the accuracy of the screw placement. For posterior column region, Ebraheim constructed cross-sections perpendicularly to the posterior column at 1.0-cm intervals. The safe anatomical pathways for screws placed at entrance points of 2.0 cm and 3.0 cm medial to the lateral acetabular margin and angled medially 45° and 15°, respectively [10]. This study did not provide the essential parameters of safe pathway from an anterior approach, which is also commonly used for complex acetabular fractures involving posterior column.

The comparisons between male and female were conducted to find the differences of the ranges of safe and effective angles, the safe screw lengths. All the female angles α in section 2, 3, 4 showed significantly greater than the male angles. It can thus be seen that to avoid the screw penetration of the hip joint, the female requires greater tilt towards the quadrilateral surface than the male. All the maximum effective angles β in section 2, 3, 4 between male and female showed no significant difference.

As the existing researches on the safe paths and safe angles at acetabulum were performed with cadaveric bone, some procedures during the measurements, including the projected area of acetabulum, the determination of the cross-section, the construction of the supplementary lines, etc., contain a certain level of artificial error. The cross-sections at fixed intervals for all sizes of pelvis might result in sections at different anatomical positions, which made the measurement results and comparisons of these cross-sections less reliable and valuable. Besides, the sample sizes of these researches are relatively low, ranging from 30 to 46, which are relatively small to evaluate the general situation of a population. Our study applied digital approaches to divide the cross-sections and measure the angles, with a relatively larger sample size, which would provide more reliable reference for acetabular surgeries.

### Limitations and shortcomings

In this study, all subjects were unrelated ethnic Han Chinese recruited from the authors’ institution. It would be interesting to conduct independent studies in other ethnic populations for comparison. In addition, due to the different medical conditions of each sample, we could not gather the complete data of each sample’s height and weight. It would be meaningful to perform a follow-up study, which expands the analysis including these parameters.

## Conclusions

By digital reconstruction and 3-dimensional measurement of a large sample of normal adult pelvic CT scans, this study obtained the safe and effective screw angles and lengths at acetabular area of the fixation route along the superior border of the arcuate line to the pubic tubercle. The screw insertion at the acetabular area for the female requires greater minimum safe angle towards the quadrilateral surface than the male. While not addressing soft tissue coverage, surgical exposure, or reduction effects of the fracture during surgeries, this 3D measurement study of safe screw pathways based on the reconstruction of pelvis provides a solid reference for the treatment of acetabular fractures.
